# Deletion of Genes Implicated in Protecting the Integrity of Male Germ Cells Has Differential Effects on the Incidence of DNA Breaks and Germ Cell Loss

**DOI:** 10.1371/journal.pone.0000989

**Published:** 2007-10-03

**Authors:** Catriona Paul, Joanne E. Povey, Nicola J. Lawrence, Jim Selfridge, David W. Melton, Philippa T. K. Saunders

**Affiliations:** 1 Medical Research Council Human Reproductive Sciences Unit, Queen's Medical Research Institute, Edinburgh, United Kingdom; 2 Sir Alastair Currie Cancer Research United Kingdom Laboratories, Molecular Medicine Centre, University of Edinburgh, Western General Hospital, Edinburgh, United Kingdom; University of Helsinki, Finland

## Abstract

**Background:**

Infertility affects ∼20% of couples in Europe and in 50% of cases the problem lies with the male partner. The impact of damaged DNA originating in the male germ line on infertility is poorly understood but may increase miscarriage. Mouse models allow us to investigate how deficiencies in DNA repair/damage response pathways impact on formation and function of male germ cells. We have investigated mice with deletions of ERCC1 (excision repair cross-complementing gene 1), MSH2 (MutS homolog 2, involved in mismatch repair pathway), and p53 (tumour suppressor gene implicated in elimination of germ cells with DNA damage).

**Principal Findings:**

We demonstrate for the first time that depletion of ERCC1 or p53 from germ cells results in an increased incidence of unrepaired DNA breaks in pachytene spermatocytes and increased numbers of caspase-3 positive (apoptotic) germ cells. Sertoli cell-only tubules were detected in testes from mice lacking expression of ERCC1 or MSH2 but not p53. The number of sperm recovered from epididymes was significantly reduced in mice lacking testicular ERCC1 and 40% of sperm contained DNA breaks whereas the numbers of sperm were not different to controls in adult *Msh2* −/− or *p53* −/− mice nor did they have significantly compromised DNA.

**Conclusions:**

These data have demonstrated that deletion of *Ercc1*, *Msh2* and *p53* can have differential but overlapping affects on germ cell function and sperm production. These findings increase our understanding of the ways in which gene mutations can have an impact on male fertility.

## Introduction

Infertility represents a major clinical problem and affects between 17 and 25% of couples in Europe [1]; in 50% of cases the problem has been attributed to the male partner. Assisted reproductive technologies such as in vitro fertilization (IVF) and intracytoplasmic sperm injection (ICSI) [2] are widely used to treat male infertility and although sperm from infertile men often have elevated levels of DNA damage this appears to have little effect on their ability to fertilize the oocyte following ICSI [3]. As a result the use of sperm from subfertile men could result in the introduction of damaged DNA of paternal origin into the embryo. A number of studies have shown that DNA damage in sperm caused, for example, by oxidative stress (in smoking fathers) can be passed from the father to the offspring following ICSI/IVF treatment [4], [5] and that this is associated with an increased incidence of childhood cancer in their offspring [6], [7]. We believe that mouse models constitute an ideal model system to investigate the origins and consequences of DNA damage in sperm. A variety of DNA repair and DNA damage response pathways are operative in male germ cells and therefore we undertook a study comparing the consequences for spermatogenesis and male germ cell DNA integrity of defects in two different DNA repair pathways and a DNA damage response pathway. Since the long-term aim is to investigate the consequences of DNA damage in sperm on early embryonic development we chose mutants with only mild to moderate defects in spermatogenesis.

Spermatogenesis is a complex, multi-step process that involves DNA replication, meiosis and DNA packaging [8]. During meiosis recombination events play an important role in creating genetic diversity among individuals within a population [9]. Recombination involves the induction of double strand breaks (DSBs), followed by crossing over between homologues and ligation of DNA molecules. Failure to introduce DSBs results in recombination and synapsis failure, leading to meiotic failure and infertility e.g. in *Spo11* knockout mice, [10], [11]. Likewise failure to repair the DNA breaks is also deleterious as demonstrated by examination of mouse knockouts of genes involved in recombination repair pathways shown to result in male infertility [12], [13].

Proteins involved in mismatch repair (MMR), nucleotide excision repair (NER), base excision repair (BER), single strand break repair (SSBR) and double strand break repair (DSB) are all expressed in the testis [14]. For example, histone variant 2AX (H2AX) is involved in the DNA damage response to DSBs [15]–[18] and acts by recruiting DNA repair factors to sites of DNA damage where it is rapidly phosphorylated resulting in formation of γH2AX foci [19]. MMR proteins are involved in the removal of errors made in DNA replication that have escaped the proof reading activity of DNA polymerase (reviewed in [20]). In the mouse MMR pathway, five proteins (MSH2 to MSH6) function as heterodimers to initiate repair activity; MSH2/MSH6 and MSH2/MSH3 are involved in repairing replicative mismatches whereas MSH4/MSH5 is a meiosis specific complex essential for processing recombination intermediates (reviewed in [21]). MSH2 is highly expressed in mouse spermatogonia and leptotene/zygotene spermatocytes [22]. *Msh2* knockout mice manifest an enhanced predisposition to skin cancer/tumorigenesis associated with UVB exposure [23], [24] but no abnormalities in spermatogenesis have been reported.

The NER pathway is responsible for the repair of UV-induced DNA damage and bulky DNA lesions. The pathway consists of an initial lesion recognition step, followed by dual incision at the sites flanking the lesion and release of a lesion-containing oligonucleotide. Repair synthesis then takes place followed by ligation (reviewed by [25]). ERCC1 (excision repair cross-complementing protein) is essential for the NER pathway, where it acts in a complex with Xpf (ERCC4) [26], [27]. Unlike most other NER proteins ERCC1 is also involved in, but not essential for, the homologous repair (HR) pathway where it removes protruding single-stranded ends adjacent to regions of homology [28]. *Ercc1* knockout male mice [29] die before the first wave of spermatogenesis due to liver failure. However introduction of an *Ercc1* transgene under the control of a liver specific promoter allows mice to survive until adulthood [30]. We have previously established that the highest level of ERCC1 expression occurs in pachytene spermatocytes and round spermatids but that adult male liver-transgene positive (*TG-Ercc1*) mice are infertile due to germ cell loss [26].

If DNA damage is not repaired or tolerated then the cells that harbour the damage are removed by the apoptosis pathway. In spermatogenesis DNA damage-induced apoptosis is p53 dependent though elimination of spermatocytes with synaptic errors appears to be p53 independent [31]. The p53 tumor suppressor protein is highly expressed in the testis and some studies have suggested a role for p53 in meiosis as it is expressed in pachytene [32] and pre-leptotene [33] primary spermatocytes. p53 is also thought to play a role in repair of DNA strand breaks: it has been shown to bind single stranded DNA and promote strand transfer between complementary strands [34] and plays a direct role in HR [35] and also in the non homologous end-joining of DSBs [36]. p53 knockout mice have been shown to exhibit less mature motile sperm than controls and this would be consistent with compromised DNA repair and apoptosis [37], [38]. In the present study we have examined testicular function in three different lines of transgenic mice with deletions in genes involved in maintaining DNA integrity in the germline. These mice have deletions in *Ercc1*, *Msh2* or *p53*.

## Materials and Methods

### Animals

The production of ERCC1 deficient mice with a liver specific Ercc1 transgene (TG-Ercc1) has been described previously [30]; animals are on a segregating Balb/c X 129Ola X C57BL/6 background. The production of the Msh2 mice (C57Bl/6 background) and p53 mice (CBA background) have been described previously by de Wind et al [39] and Clarke et al [40] respectively. All animals were bred and maintained in University of Edinburgh animal facilities under standard conditions and procedures were carried out according to Home Office regulations and the Animals and Scientific Procedures Act 1986.

### Measurement of tubule diameters

Testes were fixed in Bouins for 8 h before cutting them in half; tissues were stored in 70% (v/v) ethanol and processed into paraffin wax using standard procedures. Tissue sections (5 µm) were stained with haematoxylin and eosin; images were captured from an Olympus microscope BH2 (Olympus, UK) under a x40 lens using a video camera (Hitachi HV-C20, Japan) and were analyzed with Image Pro Plus™ software with a Stereology 5.0 plug-in (Media Cybernetics, Berkshire, UK). The software was used to trace around each section, creating an area of interest (AOI). Twenty-five fields, randomly selected by the program, within the AOI were then examined on one testis section from each animal. The software takes the average of three measured diameters per tubule. A minimum of 75 tubules per testis was measured and the frequency distribution of diameter determined.

### Immunolocalisation of caspase 3 positive cells

Testis sections (5 µm) were dewaxed in xylene and rehydrated through a series of graded alcohols. Antigen retrieval was performed using 0.01 M citrate buffer (pH 6.0). Sections were pressure cooked for 5 min at full pressure, left to stand for 20 min, and cooled under running tap water. Endogenous peroxidase activity was blocked by washing in 3% (v/v) hydrogen peroxide in methanol for 30 min at room temperature. All washes between incubations comprised 2×5 min at room temperature in Tris-buffered saline (TBS; 0.05 M Tris-HCl (pH7.4), 0.85% NaCl). Sections were incubated in normal goat serum (NGS; Autogen Bioclear UK Ltd, Wiltshire UK) diluted 1∶4 in BSA/TBS (5%, w/v) for 30 min to block non-specific binding sites. Sections were incubated overnight at 4°C with the primary antibody specific for cleaved caspase 3 (Cell Signaling Technology, Beverly, MA, USA) diluted 1∶200 in NGS/TBS/BSA. Negative control sections were incubated with blocking serum alone to confirm antibody specificity. Sections were incubated with biotinylated goat anti rabbit secondary antibody diluted 1∶500 in NGS/TBS/BSA for 30 min at room temperature. Bound antibodies were detected according to standard methods [41]. Counts of caspase positive germ cells were made on a Provis AX70 microscope (Olympus Optical, London, UK). This was achieved by counting the total number of positive cells in four testis sections per animal, at least 50 µm apart.

### Recovery of spermatozoa and sperm counts

Epididymides were placed in 500 µl of fresh Biggers, Whitten and Whittingham medium (BWW; [42]). The tissue was thoroughly minced with fine scissors and incubated at 32°C for 30 min to allow the motile spermatozoa to ‘swim up’. The motile spermatozoa fraction was removed leaving debris behind and made up to 1 ml in BWW. Aliquots were made for the sperm chromatin structure assay (see below). For sperm counts the sample was diluted in 4% paraformaldehyde (PFA) and counted using a hemocytometer. Two counts were made for each sample and the mean taken.

### Sperm Chromatin Structure Assay (SCSA)

A modified version of the SCSA method described by Evenson *et al.* 1999 was used. Comparison with other DNA strand break assays (TUNEL and COMET) has shown that the DNA denaturation measured in the SCSA is largely due to DNA strand breaks [43]–[45]. The sperm samples in BWW were adjusted to a concentration of 1–2×10^6 ^cells/ml with TNE (0.15 M NaCl, 0.1 M Tris, 1 mM EDTA pH 7.4). 100 µl of this sample was mixed with 200 µl acid detergent solution (0.1% TritonX-100, 0.15 M NaCl, 0.08 M HCl). After 30 sec, 600 µl acridine orange (AO; Sigma) diluted in AO staining solution (37 mM citric acid, 126 mM Na_2_HPO_4_, 1 mM EDTA, 0.15 M NaCl, pH 7.4 with AO added fresh to a final concentration of 6 µg/ml) was added to the sperm, mixed and incubated for 3 min at room temperature before analysing using a fluorescent assisted cell sorting (FACS) machine.

The sperm samples were analysed on a Coulter Epics XL Flow Cytometer (Beckman Coulter Ltd. High Wycombe, Bucks, UK) with a 480 nm excitation laser. Green fluorescence (from double-stranded DNA) was detected using a bandpass filter (530 nm±15) and red fluorescence (single-stranded DNA) using a longpass filter (>650 nm). The cytometer was calibrated for each run by adjusting the wild type control samples to give a mean fluorescence value (arbitrary units) of 145±10 at 675 nm and 445±10 at 525 nm. 10, 000 events (sperm cells) were read for each sample. Raw data were analysed using Flowjo Software (Tree Star Inc., Ashland, Oregon, USA). Background contamination (cells other than spermatozoa) was removed by gating. The extent of denaturation of the sperm DNA was determined by calculating the DNA fragmentation index (DFI), which is based on the ratio of denatured spermatozoa DNA (red) to total spermatozoa DNA (red/[red+green]) fluorescence (%DFI).

### Spermatocyte spread preparations

Spreads were prepared as described by Barlow et al. [46]. Briefly, testes were dissected into PBS then quickly moved into 200 µl RPMI media (Sigma) warmed to 32°C where the tunica albuginea was removed using forceps and discarded along with any large blood vessels. The remaining tubules were finely chopped using two scalpel blades to form a milky suspension. This was diluted to a final volume of 3 ml RPMI. The resulting cell suspension was transferred to a 15 ml falcon tube where the tubular remnants were allowed to settle. The non-remnant fraction was transferred to a fresh tube and centrifuged at 1000 rpm for 5 min. The pellet containing germ cells was re-suspended in 2 ml warm RPMI.

Glass slides (BDH) previously boiled in dH_2_0 and air-dried were coated in 5 drops of 4.5% sucrose solution in dH_2_O using a Pasteur pipette. A glass pipette was filled with the cell suspension and one drop was dropped onto the slides from a height of 20–30 cm. Two drops of 0.05% TritonX-100 (in dH_2_0) were added to each slide for 10 min at room temperature followed by 8 drops of fixative (2% formaldehyde, 0.02% SDS, pH 8.0) per slide and incubated for 1 h in a humidified chamber. The slides were dipped briefly six times in dH_2_0 and allowed to air dry for 5 min before storing at −70°C until use.

### Immunostaining of spread spermatocytes

Slides were defrosted by washing in PBS for 5 min and blocked with blocking buffer (5% goat serum, 0.15% BSA and 0.1% Tween-20) for 1 h at room temperature. The primary antibodies (anti-SCP3 mouse monoclonal (1∶400, Abcam, Cambridge UK) and anti-γH2AX rabbit polyclonal (1∶200, Upstate Biotechnology, MA, USA)) were diluted in blocking buffer and incubated overnight in a humidified chamber at 4°C. After three 5 min washes in PBS the secondary antibodies (goat anti-mouse Alexa-546, goat anti-rabbit Alexa-488 both 1∶500, Molecular probes, Invitrogen, Paisley, UK) were applied and incubated for 1 h at room temperature. Following another three PBS washes the slides were subsequently incubated with DAPI nuclear stain (Sigma) at 1∶1000 in PBS for 10 mins before two final PBS washes. Lastly, the slides were mounted in Permafluor aqueous mounting medium (Beckman Coulter).

For γH2AX quantification, 50 spread pachytene nuclei were analysed from each mouse and the number of foci falling on the synaptonemal complexes counted. Microscope analysis was performed on a Zeiss LSM 510 Meta Axiovert 100 M confocal microscope (Carl Zeiss Ltd, Welwyn Garden City, UK).

### Statistical analysis

Results expressed as means and standard errors of the mean were analyzed using one-way ANOVA followed by the Bonferroni *post hoc* test, using GraphPad Prism version 4 (Graph Pad Software Inc., San Diego, CA).

## Results

### Testicular phenotypes of mice lacking Ercc1, Msh2 or p53

**Figure 1:**
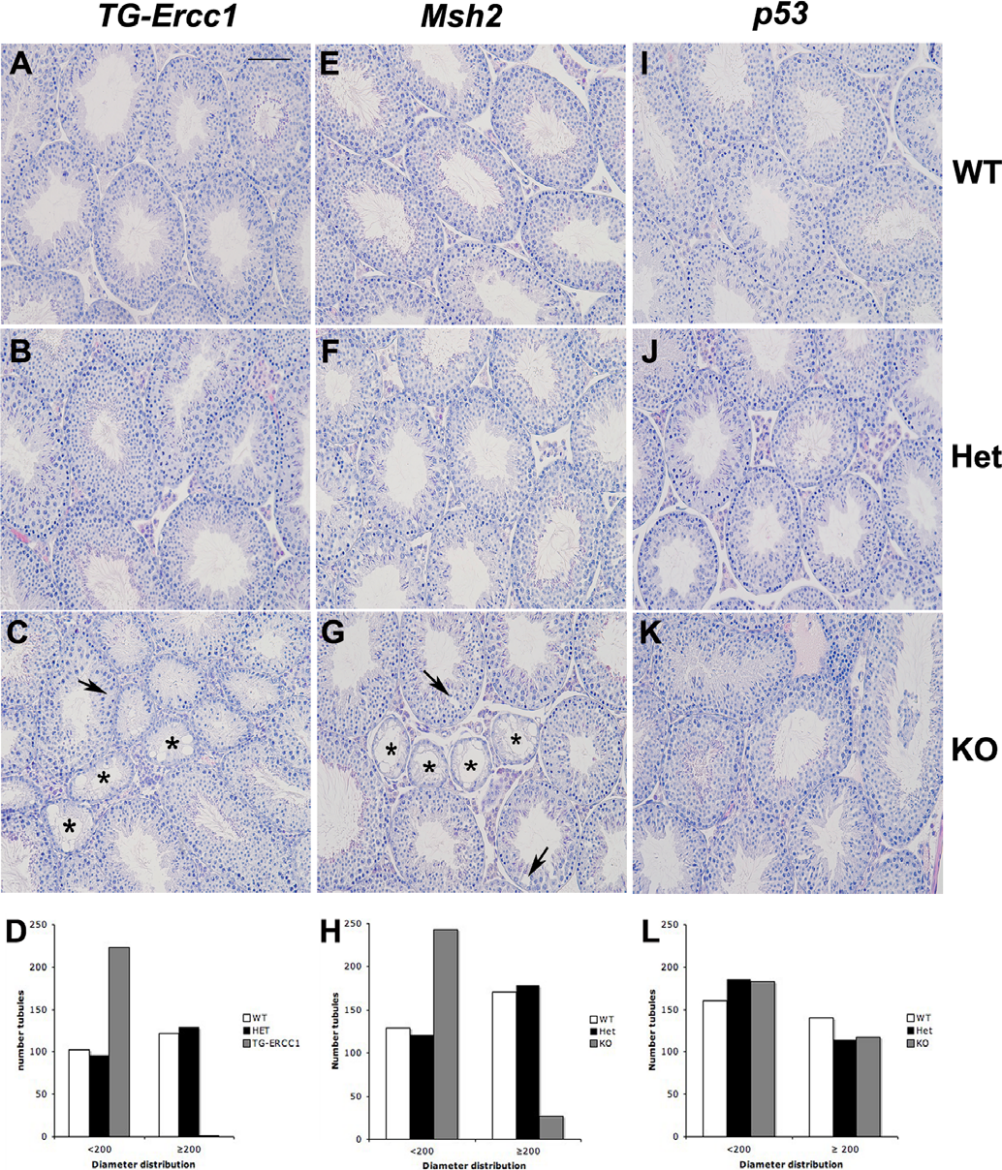
Histological evaluation of testes from adult mice. Haematoxylin and eosin staining of *Ercc1* (A–C), *Msh2* (E–G) and *p53* (I–K) testes. Sertoli cell only (SCO) tubules are highlighted with asterisks and gaps in testicular epithelium with arrows in *TG-Ercc1* −/− (C) and *Msh2* −/− (G). Bar = 100 µm. Distribution of seminiferous tubule diameters in D) *Ercc1*, H) *Msh2* and L) *p53* lines.

Examination of haematoxylin and eosin stained sections revealed disturbances in testicular architecture in *TG-Ercc1* −/− and *Msh2* −/− mice compared with control littermates (Figure 1). Consistent with our previous observations [26] *TG-Ercc1* −/− testes had reduced numbers of germ cells within the seminiferous tubules and some tubules lacked germ cells altogether (Sertoli cell only, SCO) (Figure 1C, asterisks). All phases of germ cells were observed up to and including mature elongate spermatozoa. A similar, if less pronounced testicular phenotype was observed in the *Msh2* −/− testes (Figure 1G, asterisks). In both KOs the presence of tubules with gaps in the seminiferous epithelium were observed (Figure 1 C & G arrows). Mice heterozygous for *Msh2* and *Ercc1* were comparable to their wild type littermates. Testes from *p53 −/−* mice appeared normal (Figure 1K).

### Reduction in tubule diameter in Msh2 and Ercc1 depleted testes

In *TG-Ercc1* −/− and *Msh2* −/− testes, changes in germ cell complement resulted in alterations in tubule diameters when compared to their littermate controls. In the *TG-Ercc1−/−* mice 99.5% of tubules were less than 200 µm in diameter whereas there was an equal distribution of tubules > and <200 µm in +/− and +/+ mice (Figure 1D). In the *Msh2* −/− testes the majority of tubules were <200 um (Figure 1H). Consistent with the testicular histology there was no difference in tubule diameters between testes from *p53* −/−, +/− and +/+ mice (Figure 1L).

### Increased apoptosis in Ercc1, p53 and Msh2 depleted testes

**Figure 2:**
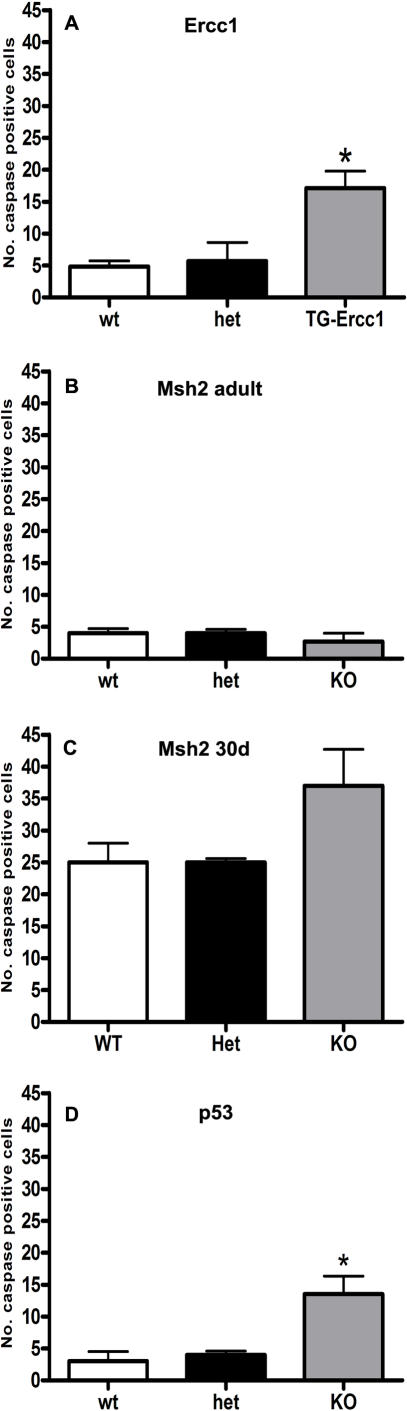
Quantification of caspase-3 positive germ cells. The total number of caspase 3 positive cells was determined in 4 sections from each mouse. An * (P<0.05) indicates significant variation compared with wild type littermates. For *TG-Ercc1* data n = 5, for *Msh2* and *p53* data n = 3.

Testicular sections were stained for cleaved caspase-3 to detect any cells that were undergoing apoptosis. In testes from *TG-Ercc1 −/−* mice a 3.5 fold increase in apoptotic germ cells was observed compared to controls (Figure 2A). A similar increase was evident in the *p53* −/− mice (Figure 2D). The majority of cells in these mutants undergoing apoptosis appeared to be spermatogonia or secondary spermatocytes and most occurred at stage XII of spermatogenesis. No increase in caspase 3-positive cells was observed in the *Msh2* −/− adults. In order to determine whether an earlier wave of apoptosis might account for the presence of SCO tubules in the adult *Msh2* −/− testes, testes from mice during the first wave of spermatogenesis (day 30) were also examined and in these a slight but non-significant increase in the number of caspase-3 positive germ cells was detected (Figure 2C).

### DNA damage in pachytene spermatocytes

**Figure 3:**
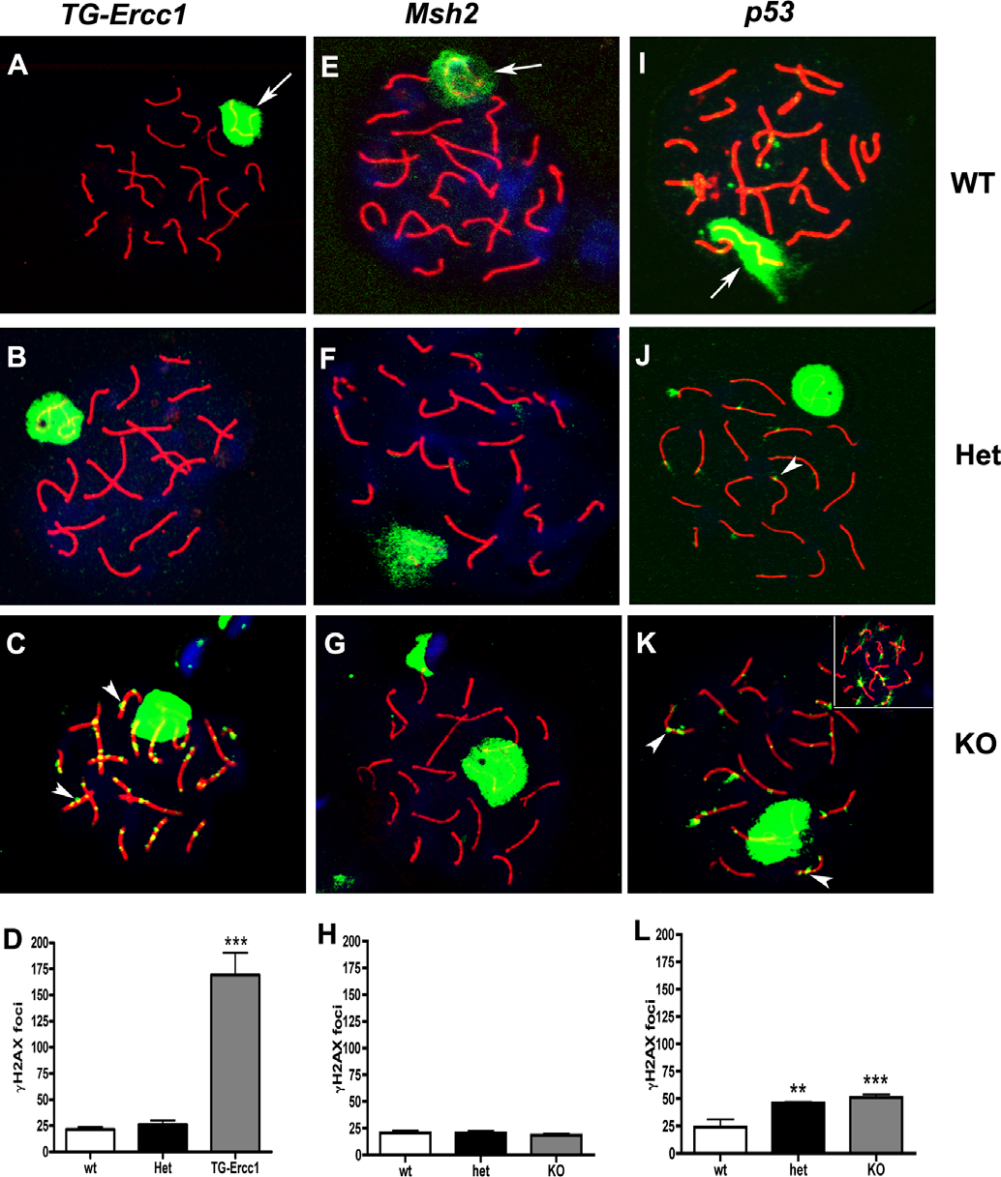
Double strand breaks in pachytene spermatocytes detected using γH2AX immunohistochemistry on germ cell spreads. Immunodetection of SCP3 (red) and γH2AX (green) in *TG-Ercc1* (A–C), *Msh2* (E–G) and *p53* (I–K) spermatocytes. The Sex body is highlighted with white arrows and foci are highlighted with arrowheads. Quantification of γH2AX foci per 50 pachytene spermatocytes per mouse in D) *TG-Ercc1*, H) *Msh2* and L) *p53* (n = 3–7). ** (P<0.01) and *** (P<0.001) indicates significant variation compared to wild type.

Germ cell spreads prepared from the testes of all three mutant lines were stained for γH2AX to detect unsynapsed DNA or double strand breaks. Co-staining with an antibody directed against the synaptonemal complex protein SCP3 was used to identify those spermatocytes at the pachytene stage of meiosis [47]. Consistent with previous publications [48] in pachytene spermatocytes from the testes of all three lines of mice intense immunopositive staining for γH2AX was localized to the sex body which contains unpaired regions of the X and Y chromosomes (Figure 3, white arrows). Variability in the numbers of unrepaired DNA lesions (γH2AX-stained foci) in pachytene spermatocytes from *TG-Ercc1 −/−* mice was observed. For example, in some spermatocytes a 600% increase in the number of γH2AX foci on the autosomes was detected (Figure 3C, white arrowheads) compared with those from +/+ or +/− animals where autosomes were on the whole γH2AX-negative. However although some spermatocytes from *TG-Ercc1* −/− were γH2AX-negative the overall quantification of foci confirmed that the numbers were significantly increased (Figure 3 D). Quantification revealed that spermatocytes from both *p53* +/− and −/− mice had a significant increase in the number of γH2AX positive foci (Figure 3J and K, summarised in L). In some *p53* −/− spermatocytes γH2AX staining was not detected on the sex body (X and Y chromosomes) (Figure 3K inset). There was no difference in γH2AX expression observed in the *Msh2* −/− mice compared to the +/− and +/+ littermates.

### Sperm counts and chromatin abnormalities

**Figure 4:**
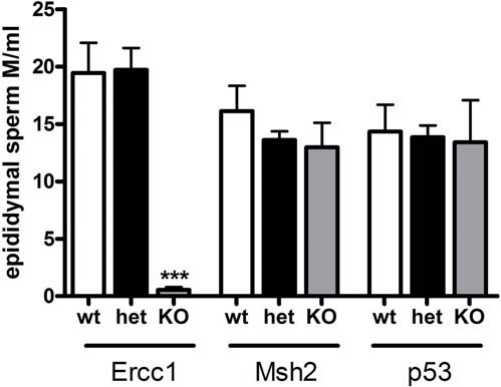
Epididymal sperm counts in *Ercc1*, *Msh2* and *p53* lines. *** (P<0.001) indicates significant variation compared with *Ercc1* wild type littermates, M = millions. For all three lines n = 4.

**Figure 5:**
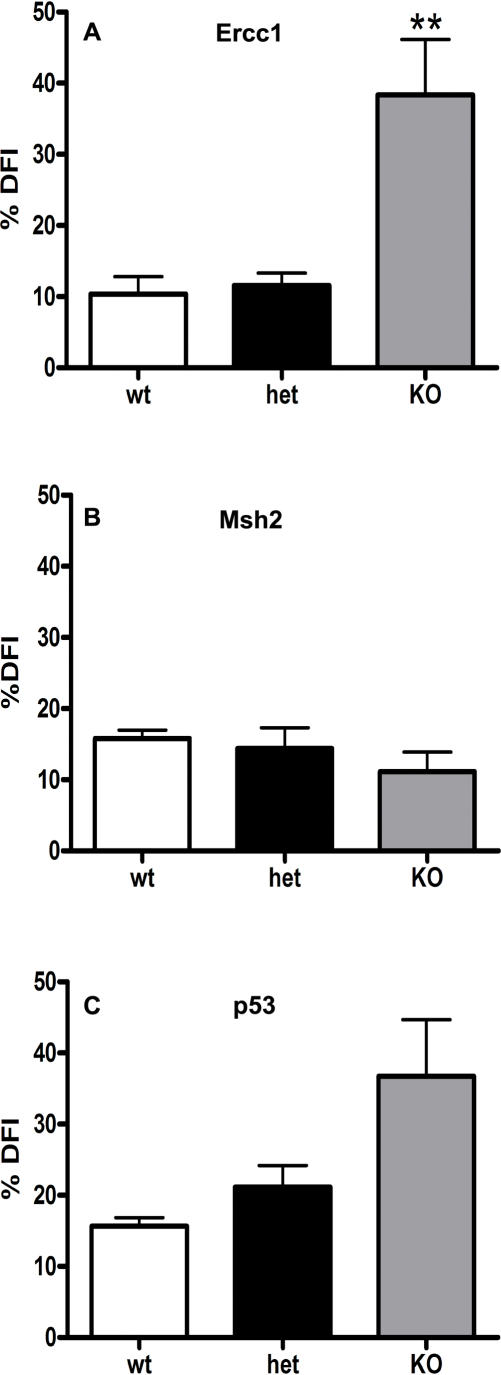
Susceptibility of epididymal sperm to acid denaturation detected by the SCSA. %DFI represents ratio of denatured sperm DNA (red) to total sperm DNA (red/[red+green]) fluorescence. ** (P<0.01) indicates significant variation compared with wild type littermates. For all three lines n = 4.

On performing sperm counts it was evident that only the *TG-Ercc1* −/− mice had a significant reduction in epididymal sperm with a 97% decrease compared with wild type littermates (Figure 4). To investigate whether there was altered chromatin structure in the sperm from the mutant lines the SCSA was used. The sperm from the *TG-Ercc1* −/− mice exhibited a significant increase (3 fold) in the percentage with DNA damage compared to controls (Figure 5A). The p53 line also showed altered sperm chromatin structure with a 2.5 fold increase (not significant) in the percentage of *p53 −/−* sperm with DNA damage over the wild type (Figure 5D). However, sperm from the *Msh2* line exhibited low percentages of sperm with DNA damage or abnormal chromatin irrespective of genotype.

## Discussion

The impact of damaged DNA originating in the male germ line is poorly understood, but may contribute to early pregnancy loss (recurrent miscarriage), placental problems and have an impact on the health of the offspring. A number of studies have shown a link between DNA damage in sperm and early embryonic failure. Spermatogenesis is a complex process that cannot, as yet, be modelled in vitro [8], involving mitotic proliferation, meiotic division and spermiogenesis, followed by release of mature sperm and taking 35 days in mice (75 days in human)[49]. Studies using infertile and subfertile mouse strains, have identified a number of genes that are essential for DNA repair, meiosis and germ cell-somatic cell interactions [50]–[54]. In the present study we compared the integrity of DNA in germ cells and sperm from mice with defects in three different genes the products of which are involved in DNA repair or DNA damage response pathways. Although all three mutants studied were on different genetic backgrounds, it is important to note that each mutant was compared against heterozygous and wild-type littermate controls on the same background.

We had previously determined that the *TG-Ercc1* −/− mice are infertile and that they produced sperm containing damaged DNA as determined by a single cell COMET assay [26]. In the present study we quantified the expression of caspase-3, one of the ‘executioner caspases’ that are known to be activated by the FAS system (reviewed in [55]) and found this to be 3.5x higher in germ cells of the *Ercc1* −/− males than in control littermates. A proportion of the seminiferous tubules lacked germ cells (SCO tubules) and no seminiferous tubules with diameters >200 nm were detected consistent with overall loss of germ cells. Using the SCSA to determine the proportion of sperm in the population with chromatin abnormalities we found this to be 3 fold higher in the mice lacking ERCC1 in their testes.

The mouse MMR pathway is involved in repairing a variety of mismatches and also participates in recombination during meiosis [56]–[58]. In mammals, five MutS homolog proteins (MSH2 to MSH6) function as heterodimers to initiate repair activity; MSH2/MSH6 and MSH2/MSH3 are involved in repairing replicative mismatches whereas MSH4/MSH5 is a meiosis specific complex essential for processing recombination intermediates [59], [60]. There are also four MutL homologs: MLH1, MLH3, PMS1 and PMS2 [13], [61]. MLH proteins are also involved in meiosis, for example, MLH1 is found at sites of meiotic crossing over [62]. Consistent with the need to correctly repair recombination events mice deficient in MSH5 or MLH1 exhibit disrupted spermatogenesis and are infertile [59], [63].

Prior to the data in the current study there had been no indication that *Msh2* −/− male mice had any disturbance in testicular function. However MSH2-MSH3 dimers have been shown to form complexes on repetitive sequences at centromeres and on the Y chromosome during meiotic prophase and in *Msh2* nulls there is a significant decrease in the accumulation of other MMR proteins (MLH3) at centromeres [64]. In common with the *TG-Ercc1* −/− the testes from *Msh2* −/− mice contained SCO tubules as well as tubules with ‘gaps’ in the epithelium. A reduced germ cell complement resulted in an increase in the proportion of tubules with diameters <200 µm although this was not associated with an increase in germ cells that were immunopositive for cleaved caspase 3. When we examined testes from 30-day-old *Msh2* −/− males a slight increase (not significant) was observed in caspase 3-positive germ cells suggesting that the presence of MSH2 may be more critical during the first wave of spermatogenesis when the process is less efficient. In addition the presence of some SCO tubules suggests that this increase in germ cell apoptosis resulted in loss of spermatogonia and therefore all subsequent germ cell types although this requires further investigation. Although there appeared to be some germ cell loss, the *Msh2* −/− mice were still fertile consistent with recovery of epididymal sperm that were only slightly, and non-significantly, reduced in number compared with normal littermates. The SCSA did not detect an increased frequency of sperm with abnormal chromatin structure in *Msh2* −/− adults which is reassuring as this assay detects altered chromatin structure arising from DNA breaks and would not be expected to reveal DNA mismatches arising from MSH2 deficiency although lesions of this type could have a significant effect on early embryonic development.

The effect of p53 deficiency on fertility appears to be mouse strain dependant; while on most backgrounds *p53* −/− mice are fertile, male infertility has been reported in mice on the 129 genetic background [65]. In a study by Embree-Ku and Boekelheide [66] spermatogenesis and fertility appear to be unaffected by the loss of p53 in mice with a C57BL/6xC3H background. However, other reports showed increased levels of sperm chromatin abnormalities and reduced fertility using mice on a pure C57BL/6 background [38]. Analysis of sperm from 129×C57BL/6 mice with the single cell COMET assay did not detect an increase in DNA damage [67]. The present study is the first to report the phenotype of *p53−/−* male mice on a CBA background. A significant number of caspase-3 positive germ cells were detected in the testes and although numbers of epididymal sperm did not appear different to wild-type littermates many of them appeared to have abnormal head structure (not shown) and there was a 2.5 fold increase in the percentage of sperm with DNA damage as determined by the SCSA. p53 has previously been implicated in the regulation of the NER pathway (reviewed in [68]). It would be of use to investigate whether there were any changes in the activity of the pathway in these mutant mice. The level of apoptosis was also increased in the *p53* −/−, which is not surprising given that we found an increase in γH2AX foci in both the *p53* +/− and the *p53* −/− and indicating that loss of p53 did not prevent apoptosis.

To gain new insight into the impact of the deletion of ERCC1, Msh2 or p53 on the integrity of DNA in germ cells we examined whether persistent DNA strand breaks were present in pachytene spermatocytes by performing immunostaining for γH2AX. Phosphorylation of histone (H) 2AX is known to occur following formation of DSBs [69] and at sites of unsynapsed chromosomes such as those found in the XY (sex) body [48]. Phosphorylated H2AX (γH2AX) appears to recruit repair proteins to sites of damage [70]. In the present study we demonstrate for the first time an increased incidence of DSBs persisting in pachytene spermatocytes recovered from *TG-Ercc1* −/− testes compared to *TG-Ercc1* +/− and +/+ littermates. This would be consistent with an important role for ERCC1 in the pachytene spermatocytes of prophase I where the highest levels of expression of the protein occur [26]. The observed increase in DSBs may also explain the increase in apoptosis, the reduction in germ cell numbers and the presence of SCOs. Furthermore the detection of DSBs in pachytene spermatocytes and the abnormal chromatin structure in mature sperm confirm that the repair functions of ERCC1 are essential for normal germ cell maturation and suggest that none of the other NER proteins can compensate for its ablation. *Ercc1* [26], its partner *Xpf* [71] and other NER genes examined [72]–[74] are all highly expressed in mouse testes. NER activity has been found in male germ cells [75] and varies between murine spermatogenic cell types [76]. Mice deficient in the NER genes *Xpa* and *Xpc* are fertile, but no detailed study of spermatogenesis has been reported [77], [78]. This suggests that the spermatogenic defects we observe in *Ercc1* deficient mice are not a consequence of the lack of the NER functions of ERCC1 but are instead the consequence of its additional repair roles, such as in HR. This role is likely to be particularly important in the repair of DSBs in meiotic crossing over. Similarly the SCO phenotype reported in mHR23B deficient mice [79] is most likely the result of loss of an additional non-NER function of mHR23B, rather than its NER lesion recognition role in combination with XPC.

A significant increase in γH2AX positive foci was also detected in pachytene spermatocytes from −/− and +/− p53 mice in addition to this a small number of *p53* −/− pachytene spermatocytes appeared to have no sex body, a stage at which the X and Y chromosomes usually have a strong γH2AX signal. This may be explained by precocious XY dissociation, which has been reported in studies investigating the effects of heat stress on mice [80], [81]. Some of the occurrences may be due to the background of the mice used as CBA strains have been found previously to have an increased incidence of univalent chromosomes in spermatocytes compared with other mouse strains [82], [83]. A number of studies have reported that the apoptotic mechanism that responds to disruption of meiosis in spermatocytes is p53-independent and that FAS (CD95) is responsible for this p53-independent pathway for germ cell elimination [84]. FAS is a transmembrane receptor protein that is capable of initiating apoptosis in response to binding its ligand, FASL (CD95L) [85]. Although DNA damage-induced apoptotic elimination of spermatocytes is reported to be p53 independent, spermatogonial apoptosis is thought to be p53 dependent [31]. In this study, the majority of dying cells in the *p53*−/− appeared to be spermatogonia suggesting that apoptosis in this case does not depend solely on p53. The increase in caspase-3 staining we observed was surprising given that it is thought that caspase-3 mediated apoptosis is thought to be entirely p53 dependant [86]. Pachytene spermatocytes from the *Msh2* line did not show elevated levels of damage in terms of γH2AX immunostaining irrespective of genotype which was expected as mismatches do not lead to DNA breaks but could however cause replication errors and mutations.

In summary we have demonstrated that p53 and ERCC1 play an essential role in DNA damage repair during spermatogenesis, that lack of MSH2 affects germ cell complement in the testis and that a deficiency in the former two genes results in the production of abnormal sperm (Table 1). This may compromise embryo development and survival or possibly result in problems in offspring later in life both of which should be further investigated. It would also be of interest to determine whether deficiencies in these genes make the testes more susceptible to insults such as heat/oxidative stress and the effect this has on their offspring.

**Table 1:** Summary of phenotypes of knockout mice and proposed role of proteins during spermatogenesis

	Ercc1 −/−	Msh2 −/−	p53 −/−
Germ cell loss	yes	yes	no
SCO tubules	yes	yes	no
Reduced tubule diameter	yes	yes	no
Increased apoptosis	yes	yes (day 30)	no
		no (adult)	
Increased DNA damage in sperm	yes	no	yes
Persistent DSBs in spermatocytes	yes	no	yes
Role of protein in spermatogenesis	Essential.	Not essential.	Surveillance.
	Ablation results in persistence of unrepaired DNA and germ cell death	A role in repair functions during 1^st^ wave of spermatogenesis; other MSH proteins more critical in adults	Ablation results in survival of more germ cells with damaged DNA.
